# Vesicles Generated during Storage of Red Blood Cells Enhance the Generation of Radical Oxygen Speciesin Activated Neutrophils

**DOI:** 10.1100/tsw.2011.25

**Published:** 2011-01-18

**Authors:** Herbert Jank, Ulrich Salzer

**Affiliations:** Max F. Perutz Laboratories, Medical University of Vienna, Austria

**Keywords:** erythrocytes, granulocytes, neutrophils, radical oxygen species, vesicles, blood transfusion

## Abstract

Erythrocytes are known to shed vesicles *in vivo*, under various conditions *in vitro*, and, with impact for transfusion medicine, during storage of red blood cell concentrates (V_sto_ vesicles). V_sto_ vesicles of blood transfusions have been shown to deliver glycosylphosphatidylinositol-linked proteins to recipient erythrocytes, to display prothrombotic activity, and to have an inhibitory effect on macrophages. The interaction of V_sto_ vesicles with and their effect on neutrophilic granulocytes has not yet been studied in detail. Fluorescentlylabeled V_sto_ and calcium-induced vesicles were preparedin order to study the uptake of labeled vesicular components by neutrophils as compared to the process of phagocytosis of zymosan using flow cytometry and confocal microscopy. The activating effect of V_sto_ vesicles on neutrophils was addressed by a luminometric assay for stimulated radical oxygen species (ROS) generation. Coincubation of vesicles and neutrophils results in a transfer of vesicular components to the cells. This uptake is different from a phagocytotic process and is enhanced upon interference with the cellular actin cytoskeleton. Preincubation of neutrophils with V_sto_ vesicles results in an enhanced ROS generation by neutrophils, which is further increased upon fMLP stimulation and during zymosan phagocytosis. The activating effect of V_sto_ vesicles on neutrophils might be due to the specific accumulation of lysophospholipidsin V_sto_ vesicles and should be considered as a possible contributor to the pathogenesis of transfusion-related acute lung injury.

## INTRODUCTION

Erythrocytes have the ability to shed hemoglobin-rich vesicles and they lose about 20% of their surface area during their life span via vesiculation[1]. The formation of these vesicles is an inherent property of the erythrocyte plasma membrane. It can be classified as an exovesiculation process but must be differentiated from the release of exosomes, which are formed within multivesicular bodies of nucleated cells. In the literature, the erythrocyte vesicles are nonuniformly addressed as microvesicles, exovesicles, ectosomes, or microparticles. A variety of conditions are known that trigger the vesiculation process *in vitro*: (a) incubation of erythrocytes with calcium and the ionophore A23187 leads to a sequence of changes at the erythrocyte membrane and to the concomitant formation of exovesicles (here further referred to as $V_{ca}$ vesicles)[2,3]; (b) exhaustion of the cellular energy reserve (ATP depletion)[4], incubation with (c) anionic or zwitterionic amphiphiles, and (d) lysophosphatidic acid (LPA)[5] also result in vesicle shedding from erythrocytes; (e) erythrocyte concentrates in blood transfusion units have long been known to contain increasing amounts of vesicles (here further referred to as $V_{sto}$ vesicles) dependent on the duration of storage[6,7]. A general characteristic of the erythrocyte vesicles is the strong depletion or even the absence of cytoskeletal components, and the relative enrichment of glycosylphosphatidylinositol (GPI)–linked proteins. Apart from the GPI-linked proteins, further lipid raft marker components (such as ganglioside M1 or stomatin) are enriched, whereas others (such as the flotillin proteins) are depleted from the vesicles[8]. However, there are considerable variations in the exact membrane composition of the various vesicle types that probably reflect the different mechanisms of vesicle generation; specifically, the difference in the mobility, the intrinsic curvature, and cytoskeletal attachment of the membrane components that lead to different component sorting during the budding process[9,10].

The importance of vesicles (of various cell types) in intercellular communication is now becoming increasingly evident. Specifically, vesicles of immune cells have been shown to play an important regulatory role in the immune system[11]. The (patho)physiologic importance of erythrocyte vesicles, however, is not so well established, yet. In inflammatory fluids of patients suffering from arthritis, erythrocyte vesicles were found as a source of the lipid mediator LPA, due to the proinflammatory action of the secretory phospholipase A2, thereby being involved in the inflammatory process[12]. LPA itself has been shown to trigger membrane alterations in erythrocytes that result in the exposure of phosphatidylserine (PS) and the release of microvesicles. These vesicles as well as the procoagulant parent erythrocytes are thereby thought to be involved in blood clotting[5]. PS exposure and procoagulant activity have also been observed in $V_{sto}$ vesicles that accumulate in transfusion units upon storage[10]. An immunosuppressive property has recently been ascribed to these vesicles since it was shown that they are taken up by macrophages, resulting in a significant inhibition of activation of these cells by zymosan A and lipopolysaccharide (LPS)[13]. On the other hand, blood transfusions rich in $V_{sto}$ vesicles are considered as a therapeutic approach in severe paroxysmal nocturnal hemoglobinuria (PNH), exploiting the ability of these vesicles to spontaneously transfer GPI-linked CD59 and CD55 to the patients' erythrocytes that lack these protective components against complement attack[14]. It was also found that recipient neutrophils increased the surface expression of these components upon blood transfusion, indicating a direct interaction with the donor vesicles and/or erythrocytes. However, the way of the uptake of these marker components and the effect on the activity of the neutrophils were not further addressed.

In this study, we investigated the influence of erythrocyte vesicles on human neutrophils. Using fluorescein isothiocyanate (FITC)–labeled $V_{sto}$ and $V_{ca}$ vesicles, we studied the uptake of label by neutrophils and compared it to the phagocytosis of zymosan. Further, the effect of $V_{sto}$ vesicles on the oxidative burst response of neutrophils upon fMLP and zymosan stimulation was studied.

## MATERIALS AND METHODS

### Cells

Blood was collected from healthy volunteers by venipuncture into BD Vacutainer vials K2E (Becton Dickinson, Franklin Lakes, NJ) and used immediately.

Neutrophils were isolated using the dextran/Ficoll Paque method described by Nauseef[15], with some modifications. Briefly, erythrocytes were removed from whole blood by dextran sedimentation. The supernatant was centrifuged for 12 min at 700 rpm, 4°C (Varifuge, Heraeus, Hanau, Germany) and contaminating erythrocytes were removed from the cell pellet by hypo-osmotic lysis (twice). The cells were washed in phosphate-buffered saline (PBS) containing 5 mM EDTA (PBSE) and separated by centrifugation on Ficoll Paque for 30 min at 2000 rpm, 4°C. After removal of the lymphocytes from the upper phase and the middle region, centrifugation was continued for 30 min. The neutrophils were washed in 1 ml PBSE, an aliquot was analyzed by the CASY cell counter (Schärfe Systems, Reutlingen, Germany), and resuspended in PBS to give a final concentration of $4.6 \times 10^7$ cells/ml. The purity was >97% and the viability usually >95% as judged by propidium iodide exclusion in the flow cytometer. Unless otherwise stated, 10 µl of this cell suspension were mixed with 50 µl of autologous plasma; 25 µl PBS; 10 µl PBS containing 9 mM $CaCl_2$, 5 mM $MgCl_2$ (PBSCM), and 7.5 mM glucose; and 5 µl vesicle suspension (or PBS as a control) in the various experiments.

Erythrocytes were isolated from whole blood by centrifugation (15 min at 4000 rpm, room temperature [Heraeus Varifuge]), the supernatant was discarded, and the buffy coat carefully removed. Erythrocytes were washed five times in 5 volumes Tris-buffered saline (TBS) (10 mM Tris HCl, 150 mM NaCl, pH 7.5).

Blood plasma was prepared from whole blood by centrifugation for 10 min at 13,000 rpm (Eppendorf table top centrifuge) to remove cellular components.

## Preparation of Erythrocyte Vesicles

- *$V_{ca}$ vesicles* — $V_{ca}$ vesicles were prepared from freshly isolated erythrocytes as described[8], with slight modifications. Briefly, 5 ml of washed erythrocytes were resuspended in 9 volumes TBS containing 1 mM $CaCl_2$ and 5 µM ionophore A 23187 (Sigma, St. Louis, MO), and incubated for 30 min at 37°C. After adding EDTA (end concentration of 5 mM), erythrocytes were pelleted (1 min at 4000 rpm, room temperature) and the supernatant was centrifuged (Beckmann Ultracentrifuge XL-70) for 30 min at 30,000 rpm, 4°C. The pellet was resuspended in 1 ml TBS and centrifuged for 30 min at 13,000 rpm, 4°C (Eppendorf table top centrifuge). After removing the ghosts, the pellet was resuspended in TBS and the remaining large vesicles removed by pelleting 30 sec at 13,000 rpm. The supernatant was centrifuged for 30 min at 13,000 rpm, 4°C and the vesicles suspended in PBS.
- *$V_{sto}$ vesicles* — $V_{sto}$ vesicles of several erythrocyte concentrates of various blood groups were prepared according to a protocol described in Salzer et al.[10]. The erythrocyte concentrates (in SAGM, leuko-depleted and filtrated) were obtained from the Red Cross in Vienna and had been stored for 40 days. Briefly, erythrocyte concentrates were diluted with 2/5 volumes of TBS to increase the volume of the vesicle-containing supernatant and the red blood cells were pelleted (P1) by centrifugation at $1850 \times g$ for 10 min. Vesicles are collected from the supernatant by $28{,}000 \times g$ for 35 min, resuspended in TBS, and subjected to several centrifugation steps to remove contaminating erythrocytes as detailed in Salzer et al.[10]. These additional centrifugation steps recover about 60–70% of the crude vesicle amount and result in a vesicle population with rather homogeneous size.

An aliquot of each vesicle preparation was fluorescently labeled as described below. Labeled and unmodified vesicle samples were suspended in PBS to give a final concentration of 40 mg vesicular protein/ml and stored in 20-µl aliquots at -80°C. Protein concentration was determined using the DC Protein Assay from BIORAD (Hercules, CA). The vesicles used in the experiments were always chosen to be compatible with the donor blood for the neutrophils in respect to the ABO and the Rhesus blood group system.

## Fluorescent Labeling

- *FITC labeling of vesicles* — Vesicles were suspended in 600 µl 0.1 M sodium hydrogen carbonate pH = 9.5 at a concentration of 10 mg vesicular protein/ml and incubated with 2 µl FITC (3 mg/ml ethanol) for 30 min at 37°C. Vesicles are pelleted (30 min at 14,000 rpm, 4°C) and washed twice in the reaction buffer, twice in PBS, and finally suspended in PBS to give a concentration of 40 mg/ml.
- *FITC labeling of zymosan* — Zymosan is suspended in 800 µl 0.1 M sodium hydrogen carbonate pH = 9.5 at 20 mg/ml and incubated with 1.3 µl FITC solution for 30 min at 37°C. Zymosan is pelleted, washed twice in the reaction buffer, twice in PBS, and finally resuspended in PBS to give a concentration of 20 mg/ml.
- *AF-633 labeling of zymosan* — Zymosan was labeled with Alexa Fluor 633 carboxylic acid, succinimidyl ester (Invitrogen Molecular Probes, Carlsbad, CA) according to the manufacturer's instructions.

## Preparation and Opsonization of Zymosan

The zymosan used for phagocytosis studies was prepared according to the protocol of Nuutila et al.[16]. Briefly, 46 mg zymosan of *Saccharomyces cerevisiae* (Sigma, St. Louis, US-MO) was suspended in 2.3 ml Hanks' Balanced Salt Solution (HBSS) by boiling for 20 min, ultrasonificated (Bandelin, Berlin, Germany) 50 cycles, 30 sec, then washed two times in HBSS, suspended in HBBS to give a concentration of 20 mg/ml, and stored at -80°C in small aliquots. Zymosan was opsonized in 25 volumes of plasma for 30 min at 37°C under constant shaking, centrifuged at 14,000 rpm for 2 min, and resuspended in PBSCM and 7.5 mM glucose to give a concentration of 2.2 mg/ml.

## Flow Cytometry

For zymosan- or vesicle-stimulation experiments, 100 µl cell suspensions ($4.2 \times 10^5$ cells) containing respective fluorescently labeled zymosan and/or vesicles as indicated were incubated for 20 or 30 min at 37°C under constant shaking. In some experiments, cells were washed three times in PBS to ensure complete removal of adhering vesicles. One milliliter BD lysing solution (Becton Dickinson) was added and the samples were further processed according to the manufacturer's instructions. Flow cytometry was performed on a FACSCalibur cytometer (Becton Dickinson) in the in-house FACS facility and analyzed using the Cell Quest Pro software. Neutrophils were gated and fluorescein label was detected in FL-1, AF633 label in FL-4.

## Luminometry

Stimulus-dependent generation of radical oxygen species (ROS) by neutrophils was determined by a luminol-based luminometric assay. The luminol (SIGMA) stock solution (100 mM in DMSO) was diluted (1:50) in 150 mM NaCl and added to the sample to give a final concentration of 100 µM. Neutrophils were suspended in PBSCM, 1 mM glucose, and autologous plasma (1:1), and preincubated with $V_{sto}$ vesicles (2 mg vesicular protein/ml) for 20 or 30 min at 37°C. To remove the vesicles, the suspension was centrifuged for 10 min at 2000 rpm and the cells were resuspended in PBS. In some experiments, cells were washed three times in PBS to ensure complete removal of vesicles; however, these additional washing steps did not essentially alter the result of the experiments. Then, 80-µl aliquots of this suspension (containing $4.2 \times 10^5$ cells) were mixed with 10 µl PBSCM, 7.5 mM glucose, and 5 µl luminol (2 mM in 150 mM NaCl), transferred to a 96-well plate, stimulated by the addition of 5 µl 100 µM fMLP

or opsonized zymosan, and immediately assayed for ROS generation at 37°C using the Multilabel Counter Victor $^{3}$V (Perkin Elmer, Waltham, MA). Light production was measured in 99 cycles with a short shaking event between each cycle to avoid sedimentation of the cells. In some experiments, superoxide dismutase (SOD) (SIGMA) was added at 4 units/100 µl to assess the contribution of the superoxide radical to the luminometric response. At least triplicate determinations for each experimental condition were done in parallel and the mean value of these measurements in arbitrary units was plotted against the time (minutes) to indicate the process of ROS production. The area under the curve was calculated by adding the 99 data points (each subtracted by the value of the background level) and used as basis for the statistical analysis of the experiments.

## Cytochrome C Assay

Superoxide generation was additionally determined by a cytochrome C reduction assay. After preincubation with vesicles or buffer alone, cells were washed three times in PBS and resuspended in PBSCM, 7.5 mM glucose, and 45 µl of 2 mg/ml cytochrome C (SIGMA) to give 90 µl of the standard cell density. To half of the samples, 4 units of SOD were added. Then, 10 µl of buffer alone, of 50 µM fMLP, or of opsonized zymosan (22.2 µg) were added and incubated for 15 min at 37°C. The absorbance at 550 nm was determined as a measure for superoxide anion generation.

## Inhibition of Actin Polymerization

Neutrophils were suspended in PBSCM, 7.5 mM glucose with/without 5 µM cytochalasin D, and incubated for 1 h at 37°C. The cells were pelleted for 10 min at 2000 rpm and resuspended in PBS. Then, 5 µl of the cell suspension ($4.2 \times 10^{5}$ cells) were mixed with 50 µl autologous plasma, 30 µl PBS, 10 µl PBSCM, 7.5 mM glucose, and 5 µl FITC-labeled $V_{sto}$ vesicles (40 mg vesicular protein/ml) and incubated for 30 min at 37°C. The samples were further processed for flow cytometric analyses as indicated above. The inhibitory effect of cytochalasin D on zymosan phagocytosis was used as a positive control. Therefore, 5 µl of the cell suspension ($4.2 \times 10^{5}$ cells) were mixed with 50 µl autologous plasma, 35 µl PBS, 10 µl opsonized, FITC-labeled zymosan (22.4 mg/ml in PBSCM, 7.5 mM glucose) and incubated as above.

## Confocal Microscopy

We mixed $2.1 \times 10^{5}$ neutrophils in 17.5 µl PBS with 25 µl autologous plasma and stimulated with 5 µl opsonized, AF633-labeled zymosan (11.1 µg zymosan in PBSCM, 7.5 mM glucose) and/or 2.5 µl FITC-labeled vesicles and incubated 20 min at 37°C. Then, 25 µl of this suspension were allowed to sediment on polylysine-coated cover glasses for another 20 min. The cells were fixed with 3% paraformaldehyde for 30 min and embedded in Mowiol-DABCO embedding media. The samples were analyzed using the Laser Scanning Microscope 510 Meta (Zeiss, Oberkochen, Germany) of the in-house bio-optics facility.

## Statistical Analysis

The area under the curve (= integrated luminometric output) and the absorbance at 550 nm, each corrected for the background, were the numerical data points for the analyses of the luminometric and the cytochrome C experiments, respectively. In order to compare and statistically analyze different experiments in spite of the day-to-day variation in activity of freshly prepared neutrophils from different donors, we normalized each set of data to the sample without vesicle preincubation and without SOD, which was set to 100%. The statistical significance was calculated by the Student's t-test and the respective *p* values for each condition are given in Table1.

**TABLE 1**
**Preincubation with $V_{sto}$ Vesicles Enhances ROS Generation**

| | Luminescence | | | | Cytochrome C | | | |
|---|---|---|---|---|---|---|---|---|
| $V_{sto}$ | - | - | + | + | - | - | + | + |
| SOD | - | + | - | + | - | + | - | + |
| Control | 100 | 89 (5) | 128 (10)** | 99 (9)* | 100 | 24 (1)*** | 145 (8)** | 57 (14)** |
| fMLP | 100 | 76 (3)*** | 141 (10)*** | 63 (20)** | 100 | 22 (5)*** | 128 (6)* | 50 (17)* |
| Zymosan | 100 | 85 (1) *** | 143 (10)** | 116 (10) | 100 | 25 (4)*** | 132 (6)** | 52 (11)** |

The numbers are the mean values in percent of the luminescent output (luminescence) or the absorbance at 550 nm (cytochrome C), with each dataset being normalized to the $–V_{sto}$ and –SOD condition (SEM is given in brackets). The paired datasets, +/- vesicles and +/- SOD, are statistically compared, respectively, and statistical significance is denoted as follows: $^*p < 0.05$, $^{**}p < 0.01$, and $^{***}p < 0.001$. The mean increase in ROS generation relative to control for fMLP is 4.1-fold and 1.4-fold, and for zymosan 34-fold and 2.2-fold in luminescence and cytochrome C reduction, respectively. The number of experiments (n) is 3 for all cytochrome C experiments and for all luminescence +SOD experiments, 9 for the luminescence FMLP experiments (+/- vesicles), 10 for the luminescence zymosan experiments (+/- vesicles), and 28 for the luminescence control experiments (+/- vesicles).

## RESULTS

To study the interaction of erythrocyte-derived vesicles with neutrophils, we used two different types of vesicles: the well-characterized vesicles obtained by the calcium/ionophore method ($V_{ca}$) and the clinically important vesicles that accumulate in stored red blood cell concentrates ($V_{sto}$). Flow cytometric analyses revealed that coincubation of FITC-labeled $V_{sto}$ vesicles and neutrophils in autologous plasma at 37°C resulted in a considerable uptake of fluorescent label by the neutrophils; the uptake was even more pronounced in the presence of PBS instead of plasma (Fig. 1). Repeated washing of the cells after the incubation did not significantly remove the label, thereby indicating that staining of the cells was not merely due to adherence of vesicles. The relative increase in mean fluorescence intensity in the presence of buffer instead of plasma was 95 ± 24% (n = 4), indicating that a fraction of the label is absorbed by some component of the plasma. The same results were obtained when $V_{ca}$ vesicles, freshly prepared from autologous erythrocytes, were used instead of $V_{sto}$ vesicles. Even under conditions mimicking the physiological cell counts in blood with about an 1000-fold excess of erythrocytes over neutrophils, the transfer of fluorescent label from vesicles to neutrophils was still significant (data not shown).

Next, we addressed the question whether there is an interdependence between the uptake of vesicular components and phagocytosis of zymosan. Zymosan was labeled with the red fluorescent dye AF633 (Zym-AF) in order to be able to observe zymosan phagocytosis and vesicular uptake independently. Coincubation of neutrophils with Zym-AF and FITC-$V_{ca}$ did not result in an altered uptake of one of the labels as compared to controls where neutrophils were incubated with zymosan or vesicles alone (Fig. 2), indicating that the uptake of vesicular components and phagocytosis of zymosan are independent processes.

The independence of phagocytosis and the uptake of vesicular components/label were also supported by confocal microscopic analyses. Neutrophils that had been incubated with FITC-$V_{ca}$ revealed an uneven staining with sometimes patchy or granular appearance. The staining was not limited to the plasma membrane or the phagosome, but rather spread throughout the whole cell (Fig. 3A). In contrast, Zym-AF was confined to one/several phagosome(s) within the cell and there is hardly any overlap with vesicle-derived FITC label in dual label experiments (Fig. 3B–D).

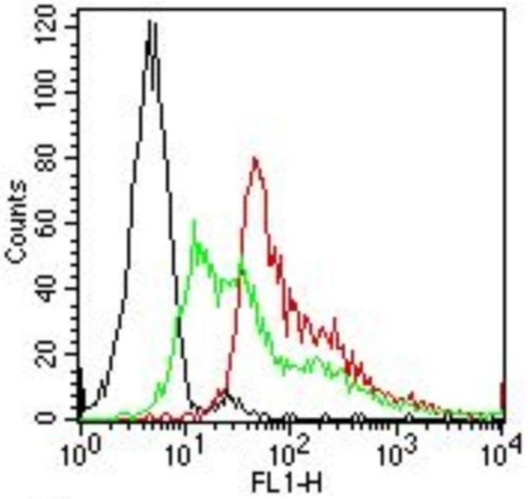

**FIGURE 1.** Uptake of labeled components from erythrocyte vesicles by neutrophils. We mixed 5 µl neutrophil suspensions ($4.2 \times 10^5$ cells) with 90 µl autologous plasma (green line) or PBS (red line) and 5 µl FITC-labeled $V_{sto}$ vesicles (40 mg vesicular protein/ml PBS), incubated for 30 min at 37°C, and immediately processed for flow cytometry (FACSCalibur) to analyze the uptake of FITC-labeled components by the cells. A representative histogram of the fluorescence intensity distribution (channel FL-1) is shown. The black line is for control neutrophils incubated in absence of labeled components.

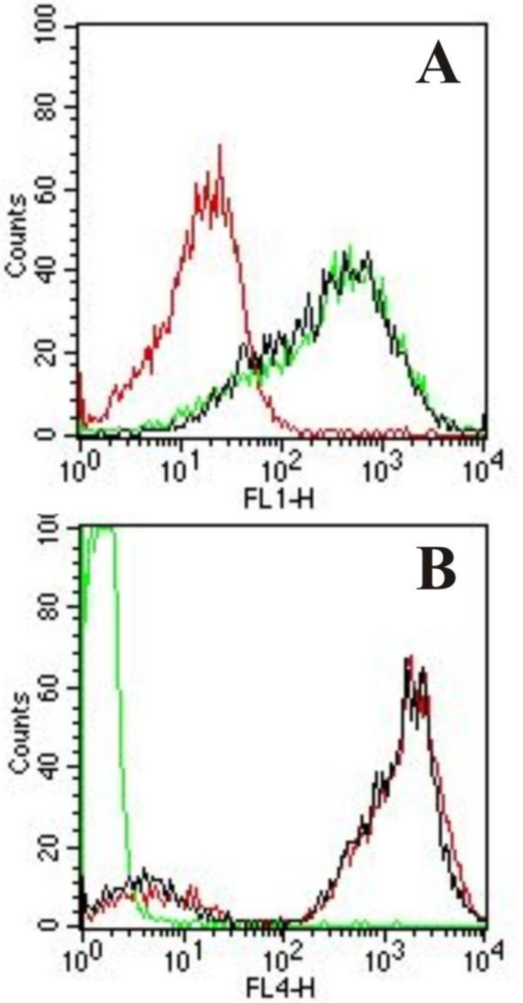

**FIGURE 2.** Vesicular uptake and phagocytosis of zymosan are independent processes. We mixed 5 µl neutrophil suspensions ($4.2 \times 10^5$ cells) with 80 µl autologous plasma and 5 µl FITC-labeled $V_{ca}$ vesicles (40 mg vesicular protein/ml PBS) (green line) or 10 µl opsonized, AF633-labeled zymosan (22.4 mg/ml PBSCM, 7.5 mM glucose) (red line), or both (black line) (respective buffers were added to the former two samples to give 100 µl), and incubated for 30 min at 37°C followed by flow cytometric analysis. Histograms of the distribution of FITC-label (A, channel FL-1) and AF633 label (B, channel FL-4) are shown indicating the independence of uptake of vesicular components and phagocytosis of zymosan.

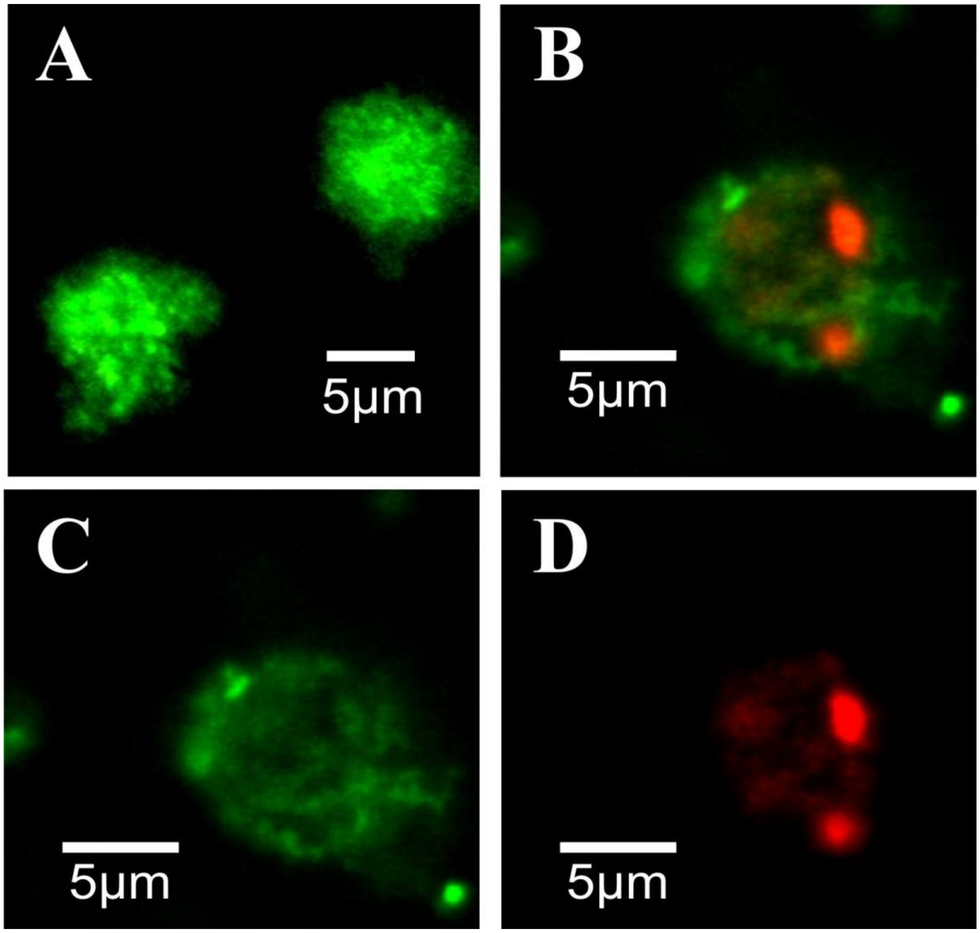

**FIGURE 3.** Difference in subcellular localization of vesicle-derived components and phagocytosed zymosan. Neutrophils were incubated with FITC-labeled $V_{ca}$ vesicles alone (A) or together with AF633-labeled zymosan (B–D) and processed for confocal microscopy analysis as described in the *Materials and Methods* section. Representative images of the dual-label experiments are shown with an overlay of the red and the green channels (B), and the respective single-channel recordings (C, D).

Further, we asked whether the cellular cytoskeleton is involved in the uptake of components from vesicles using cytochalasin D, a potent inhibitor of actin polymerization known to interfere with the phagocytic process in neutrophils. Expectedly, the uptake of FITC-labeled zymosan was decreased upon pretreatment of neutrophils with cytochalasin D (Fig. 4B). Conversely, the uptake of labeled components from $V_{sto}$ vesicles by neutrophils was increased (Fig. 4A). Using the mean fluorescence intensity of the FACS data as a quantitative measure for the cytochalasin D–dependent modulation, the uptake of label from vesicles was found to be increased to 178 ± 35%, whereas zymosan phagocytosis was decreased to 54 ± 19% (n = 3). This indicates that an intact phagocytic potential is not required for the uptake of labeled components from vesicles. Rather, some sort of nonspecific transfer has to be assumed that is enhanced upon inhibition of the actin polymerization in neutrophils.

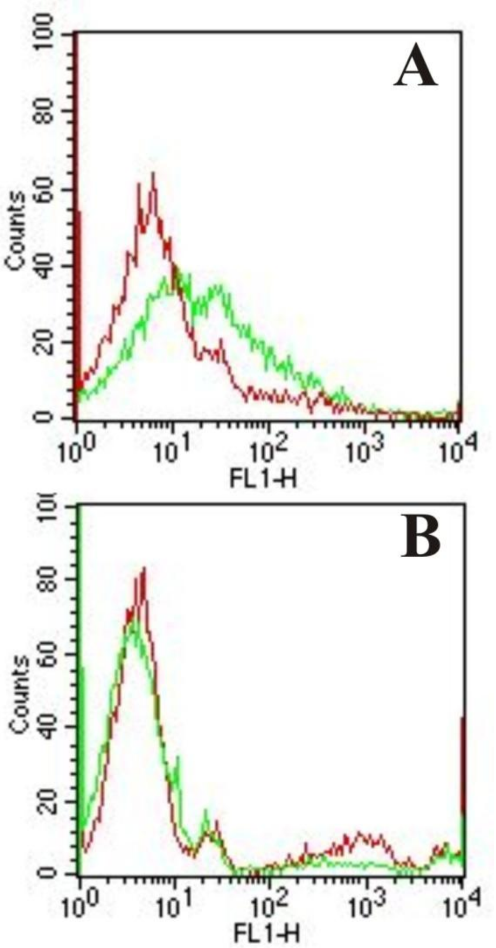

**FIGURE 4.** Uptake of vesicular components by neutrophils is increased upon inhibition of actin polymerization. To inhibit actin filament polymerization, neutrophils were suspended in PBSCM, 7.5 mM glucose with (green line) or without (red line) 5 µM cytochalasin D, and incubated for 1 h at 37°C. Cells were pelleted, resuspended, and incubated in autologous plasma, buffer, and FITC-labeled $V_{sto}$ vesicles (A) or opsonized, FITC-labeled zymosan (B) as described in the *Materials and Methods* section. Histograms of the distribution of fluorescent label in the cell population (FL-1 channel) reveal an cytochalasin D–dependent increase in the uptake of vesicular components (A) and an inhibition of zymosan phagocytosis (B).

The fact that neutrophils take up membrane components from erythrocyte vesicles upon coincubation urges the question whether this has an impact on the activity of neutrophils. Generation of ROS is a hallmark of neutrophilic activity associated with phagocytosis or stimulation by the bacterial fMLP peptide or the nonspecific neutrophilic activator PMA. Using a luminol-based luminescence assay, we asked whether erythrocyte vesicles influence the oxidative burst response during zymosan phagocytosis and upon stimulation by PMA. Unlabeled vesicles were used in these experiments to exclude the possibility that the label itself is responsible for some of the observable effects. The oxidative burst of neutrophils was reduced in the presence of $V_{ca}$ vesicles, both during zymosan phagocytosis and upon PMA stimulation. It is likely that this reduction in the oxidative burst response is due to the presence of the radical scavenging enzymes SOD and catalase, which are highly abundant in in the lumen of the vesicles. In line with this interpretation was the finding that the reduction in the oxidative burst was even more pronounced in experiments where the $V_{ca}$ vesicles were substituted by erythrocytes of the same optical density (data not shown). However, when the cells were preincubated with (unlabeled) $V_{sto}$ vesicles, washed to completely remove the vesicles, and then stimulated with fMLP or zymosan, a specific increase in the luminometric response was observed (Fig. 5B and C). Taking the area under the luminometric curve as a quantitative measure, the mean vesicle-specific increase for both stimulations was about 40% (Table 1). A considerable luminometric response was also observed in the absence of stimulation, indicating that the procedure itself triggers some sort of neutrophil activation. Again, when cells were preincubated with $V_{sto}$ vesicles, this stimulation-independent activation was increased (Fig. 5A). Since luminol is known to react with various ROS[17,18], the experiments were also performed in the presence of SOD (broken lines in Fig. 5) to estimate the relative contribution of the superoxide anion

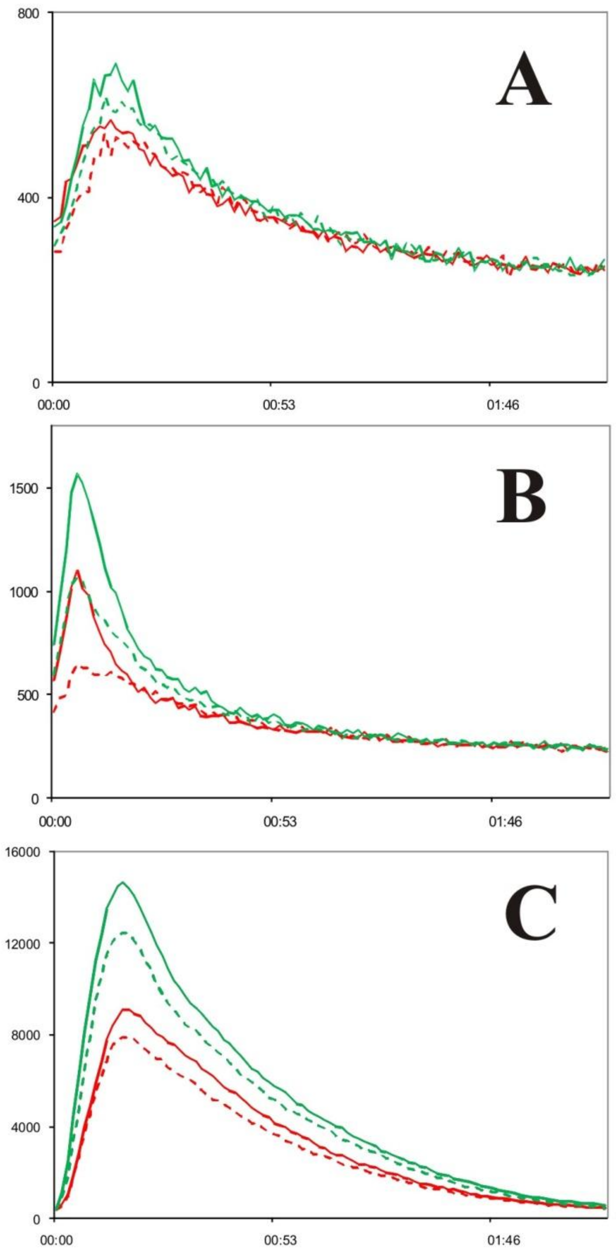

**FIGURE 5.** Preincubation with $V_{sto}$ vesicles enhances stimulus-dependent ROS generation of neutrophils. Neutrophils were preincubated with $V_{sto}$ vesicles (green lines) or buffer alone (red lines) for 30 min at 37°C. Cells were pelleted, washed three times in PBS to remove the vesicles, resuspended in luminol-containing buffer without (solid lines) or with (broken lines) SOD, left unstimulated (A) or stimulated with fMLP (B) or opsonized zymosan (C), and immediately assayed for ROS generation by luminometry as described in the *Materials and Methods* section. The luminometric output (y axis) is in arbitrary units and the time (x axis) in hours:minutes.

to the luminometric response. The fraction of superoxide (relative to other ROS) generated upon fMLP stimulation is significantly higher than during zymosan phagocytosis. This effect is even more pronounced upon preincubation with vesicles (Table 1). The vesicle-specific increase in neutrophil activity was corroborated by experiments with the complementary cytochrome C assay. In line with the high specificity of the reduction of cytochrome C for superoxide, all effects were strongly diminished in the presence of SOD (Table 1).

## DISCUSSION

In this study, we investigated the interaction of erythrocyte-derived vesicles and neutrophils, and the effect of the vesicles on neutrophilic activity. Coincubation results in a transfer of labeled components from FITC-labeled vesicles to neutrophils. Staining of the cells is not due to mere adherence of vesicles and it also takes place in the presence of a large excess of erythrocytes. The transfer from vesicles is significantly larger than from FITC-labeled erythrocytes (of the same surface area) (data not shown). In principal, there exist several possibilities for the observed transfer of vesicular components/label: (1) an active phagocytic process of whole vesicles, (2) pinocytosis of vesicular components, and (3) transfer of components via nonspecific membrane exchange processes. Several lines of evidence speak against the possibility that neutrophils ingest erythrocyte-derived vesicles by a phagocytic process. First, the uptake of label from vesicles is not influenced by zymosan phagocytosis and vice versa (Fig. 2). Second, in contrast to zymosan phagocytosis, the uptake of labeled components from vesicles was not inhibited by cytochalasin D (Fig. 3). Third, confocal microscopy revealed a diffuse staining of the granulocyte plasma membrane and cytosol by vesicle-derived FITC label, but hardly any overlap with Zym-AF in phagosomal structures in dual label experiments (Fig. 3). Fourth, uptake of label is decreased in the presence of plasma as compared to buffer (Fig. 1A), indicating that complement does not play a significant role in the uptake mechanism. In contrast, it seems that some plasma component inhibits this process (presumably serum albumin through adsorption of labeled components). These findings clearly indicate the independence of zymosan phagocytosis and uptake of label from vesicles by neutrophils.

A potential pinocytotic uptake – conceivably an indirect uptake mechanism following a preceding transfer of vesicular components to some fluid-phase component in the plasma – is not likely since the transfer of label from vesicles does not increase upon fMLP stimulation (data not shown), a feature characteristic for neutrophilic fluid pinocytosis[19]. In view of our data, we assume that nonspecific transfer of membrane components is the most likely reason for the uptake of label from vesicles. GPI-linked proteins and glycolipids are known to be enriched in erythrocyte vesicles. It is therefore likely that these components are the predominant FITC-labeled entities in our labeling procedure of vesicles and their transfer to neutrophils accounts for the major fraction of the observed uptake of fluorescent label by these cells. Cell-to-cell and vesicle-to-cell transfer of GPI-linked proteins is already a well-documented phenomenon. Sloand et al. show the transfer of GPI-linked CD55 and CD59 from donor cells/vesicles to recipient erythrocytes upon blood transfusion in PNH patients[14]. In this study, GPI-linked protein transfer to recipient granulocytes was also observed. The result of our label transfer experiment in the presence of a large excess of erythrocytes over neutrophils (not shown) is in line with this observation and indicates that a transfer of components from vesicles to neutrophils may also take place *in vivo*.

The exact mechanism of neutrophil-vesicle interaction still remains to be elucidated, however, interestingly, the uptake of vesicular components is increased upon interference with the actin polymerization machinery in neutrophils (Fig. 4). This finding may indicate that neutrophils have a membrane skeleton–dependent tendency to minimize membrane component transfer from vesicles (or other blood cells), while circulating in the blood vessel system probably in order to prevent accidental activation events.

Neutrophil activity must be tightly regulated in order to keep the balance between maximal host defense and minimal collateral host damage. Priming plays an important role in neutrophilic regulation, including direct effects on integrin expression, cell polarization, and deformability, and indirect effects by enhancing the response to a secondary stimulus. Many physiological and pharmacological substances are now known to act as priming agents, revealing a spectrum of different priming modes[20]. Blood transfusion units have also been recognized to contain a priming activity and prolonged storage increases this effect. Testing for the up-regulation of CD11b and fMLP-induced IL-8 production in neutrophils, Cardo et al. recently investigated the priming effect of various fractions of stored blood cell concentrates, showing that a considerable part of the priming activity was not red blood cell associated, but resided in the supernatant and could be removed by centrifugation[21]. Thus, microvesicles (in our terms $V_{sto}$ vesicles) were concluded to be the carriers of this effect. Our data are in line with these findings and directly show that isolated $V_{sto}$ vesicles activate neutrophils for enhanced ROS production upon fMLP stimulation and during phagocytosis (Fig. 5). Per definition, our data indicate an activating rather than a priming effect on neutrophils since the vesicles alone induce some extra activity, even in the absence of an additional stimulation (Table 1). Primed neutrophils are thought to be key players in the pathogenesis of transfusion-related acute lung injury (TRALI). Immobilization of activated neutrophils in the pulmonary capillaries seems to induce endothelial damage, fluid leakage, and the progressive development of lung edema[22]. The risk of prolonged storage of erythrocyte concentrates may be considered in this respect since $V_{sto}$ vesicles accumulate in the concentrates in a time-dependent manner[10]. However, in contrast to their priming/activating effect on neutrophils, $V_{sto}$ vesicles have recently been described to inhibit the activation of macrophages by zymosan and LPS[13]. Hence, it is conceivable that $V_{sto}$ vesicles may contribute to the immunosuppressive properties attributed to blood transfusions[13] as well as to the pathology of TRALI patients in dependence on the immunological status of the transfusion recipient.

It is known that lysophosphatidylcholine and lyso-PAF, two lipids that prime neutrophils, accumulate in stored red blood cell concentrates[23,24,25]. Lysophosphatidylcholine is thought to be generated by the action of soluble phospholipase A2 (sPLA2) on the exoplasmic membrane leaflet of the stored erythrocytes. Storage-associated cellular changes, such as increased cytosolic calcium or elevated PS exposure, are conditions that render the erythrocyte membrane more susceptible to sPLA2 action[26]. However, why is the priming/activating effect of stored red blood cell units considerably associated with the vesicles[21], although they represent only a minute fraction of the total particle mass? Two factors may be considered. On the one hand, phospholipid asymmetry between the outer and the inner membrane leaflet is not maintained in vesicles, which leads to increased PS exposure and thereby may render vesicles a preferred target for sPLA2[12]. On the other hand, $V_{sto}$ vesicles may accumulate lysophospholipids present on the red blood cell during the process of membrane budding prior to the vesicle fission event. Sorting of membrane components is associated with membrane budding and is the reason for the difference in membrane composition between vesicles and parent cells[8]. The intrinsic molecular shape is assumed to be a major determinant in this sorting process, with conically shaped components accumulating in outward-bending membrane regions[9]. Cleavage of the fatty acid at the sn-2 position changes a rod-shaped phospholipid into a cone, indicating that lysophospholipids may specifically accumulate in shed vesicles and thereby transform the vesicles to major carriers of the priming activity in blood transfusion units.

## ACKNOWLEDGMENTS

We thank the company Innutec for financial support and Thomas Sauer for helpful technical assistance.
